# Meta-analysis of the effect of perioperative intravenous lidocaine on return of gastrointestinal function after colorectal surgery

**DOI:** 10.1007/s10151-019-1927-1

**Published:** 2019-02-05

**Authors:** C. Cooke, E. D. Kennedy, I. Foo, S. Nimmo, D. Speake, H. M. Paterson, N. T. Ventham

**Affiliations:** 1 Department of Colorectal Surgery, Western General Hospital, Edinburgh, UK; 2 Department of Anaesthesia, Western General Hospital, Edinburgh, UK; 3 Department of Colorectal surgery, Western General Hospital, University of Edinburgh Academic Coloproctology, Crewe Road South, Edinburgh, EH4 2XU UK

**Keywords:** Intravenous lidocaine, Colorectal surgery, Ileus, Laparoscopic

## Abstract

**Background:**

Return of normal gastrointestinal (GI) function is a critical determinant of recovery after colorectal surgery. The aim of this meta-analysis was to evaluate whether perioperative intravenous (IV) lidocaine benefits return of gastrointestinal function after colorectal resection.

**Methods:**

A comprehensive search of Ovid Medline, PubMed, Embase, Cochrane library, and clinicaltrials.org was performed on 1st July 2018. A manual search of reference lists was also performed. Inclusion criteria were as follows: randomized controlled trials (RCTs) of intravenous (IV) lidocaine administered perioperatively compared to placebo (0.9% saline infusion) as part of a multimodal perioperative analgesic regimen, human adults (> 16 years), and open or laparoscopic colorectal resectional surgery. Exclusion criteria: non-colorectal surgery, non-placebo comparator, children, non-general anaesthetic, and pharmacokinetic studies. The primary endpoint was time to first bowel movement. Secondary endpoints were time to first passage of flatus, time to toleration of diet, nausea and vomiting, ileus, pain scores, opioid analgesia consumption, and length of stay.

**Results:**

One hundred and ninety one studies were screened, with 9 RCTs meeting inclusion criteria (405 patients, four laparoscopic and five open surgery studies). IV lidocaine reduced time to first bowel movement compared to placebo [seven studies, 325 patients, mean weighted difference − 9.54 h, 95% CI 18.72–0.36, *p* = 0.04]. Ileus, pain scores, and length of stay were reduced with IV lidocaine compared with placebo.

**Conclusions:**

Perioperative IV lidocaine may improve recovery of gastrointestinal function after colorectal surgery. Large-scale effectiveness studies to measure effect size and evaluate optimum dose/duration are warranted.

**Electronic supplementary material:**

The online version of this article (10.1007/s10151-019-1927-1) contains supplementary material, which is available to authorized users.

## Introduction

Colorectal resection causes an unavoidable cessation of normal gastrointestinal (GI) function in every patient; hence, the return of GI function is a critical determinant of recovery [1, 2]. Modern minimally invasive techniques and multimodal “enhanced recovery” programs have reduced the historically high prevalence of delayed return of GI function associated with open colorectal surgery [3, 4]. Despite this, return of GI function after colorectal resection can lag behind other aspects of recovery such as mobilization and pain control [5]. A prolonged delay in return of GI function (commonly known as postoperative ileus) is characterized by inability to resume normal diet, vomiting, abdominal distension, and absolute constipation, requires active supportive management [intravenous (IV) fluids, anti-emetics, nasogastric intubation], and results in longer hospital stay with a substantially poorer patient experience. Recovery of GI function is important to patients and surgeons alike and was identified as a key research focus in a recent research prioritization exercise undertaken jointly between patients and the Association of Coloproctology of Great Britain and Ireland [6, 7].

Perioperative IV lidocaine has well-established anti-inflammatory and opioid-sparing analgesic properties [8–10]. There are also data to suggest a beneficial effect on return of GI function following abdominal surgery. However, interpretation of the existing literature is challenging, as it includes a variety of operations, access techniques and perioperative management protocols [11, 12]. Furthermore, despite the existence of validated consensus-derived composite endpoint definitions of return of GI function (GI-2, GI-3) [2, 13, 14], many studies of perioperative IV lidocaine report a variety of sub-optimal univariate endpoints to measure GI recovery.

This study updates existing meta-analyses of perioperative IV lidocaine by inclusion of new data and seeks to limit study heterogeneity by focusing on return of GI function following colorectal surgery.

## Materials and methods

### Literature search

The study was placed prospectively on the International Prospective Register of Systematic Reviews (PROSPERO) register [CRD42016049847]. A comprehensive search of Ovid Medline, PubMed, Embase, Cochrane library, and clinicaltrials.org was completed on 5th September 2018. A manual search of reference lists was also performed. The following search strategy was used: (colorectal surgery OR colectomy OR colon OR colonic OR bowel) AND (intravenous lidocaine OR intravenous lignocaine OR lidocaine infusion OR lignocaine infusion OR IV lidocaine OR IV lignocaine OR I.V lidocaine).

### Inclusion criteria

Randomized controlled trials (RCTs), human adults [> 16 years], open or laparoscopic colorectal resectional surgery.

### Exclusion criteria

Non-colorectal surgery, non-placebo comparator, children, non-general anaesthetic, and pharmacokinetic studies.

### Intervention and comparator

Intravenous lidocaine administered perioperatively was compared to placebo (0.9% saline infusion) as part of a multimodal perioperative analgesic regimen.

### Data extraction

Full-text randomized control trials meeting inclusion criteria were reviewed by two independent researchers (EK/CC). A proforma was used to extract relevant information: data presented as mean and standard deviation were extracted directly, whereas non-parametric results (median and interquartile range) were converted using previously described techniques. For skewed data, the median was used instead of the mean [15].

### Primary outcome

Since none of the included studies reported the validated GI-2 or GI-3 definitions of GI function, the primary outcome was time (hours) to first bowel movement (various phrases “bowel function”, “defecation”, and “bowel motion” were used in the included studies and we have assumed them to mean the same thing, i.e., defecation).

### Secondary outcomes

#### Return of GI function

- Time to first passage of flatus (hours).
- Time to toleration of diet (hours).
- Incidence of postoperative ileus.
- Incidence of nausea and vomiting.

#### Pain

- Numerical pain score at rest at 24 h (score 0–10, 0 = no pain, 10 worst imaginable pain, alternative methods converted to 0–10 range).
- Numerical pain score on movement at 24 h (0–10 as above).
- Opioid consumption over first 24 h after surgery (milligrams and morphine equivalent doses).
- Total opioid consumption (milligrams).

#### Other

- Length of stay (hours).

### Subgroup analyses

A predefined subgroup analysis was performed for open and laparoscopic surgery.

### Bias and quality assessment

Overall quality and potential bias were assessed using a previously described 15-point scale adapted from criteria described by Chalmers and Jadad, with a threshold score of ≥ 12 for high quality (Table 1) [16, 17]. A sensitivity analysis was conducted for the primary endpoint by excluding each study.

**Table 1:** Details of trials included in meta-analysis

Author	Number of participants	Operation	Intervention	Protocol	Additional analgesia	Modified quality score
IV Lidocaine versus placebo as part of a multimodal analgesic regimen						
Laparoscopic						
Elhafz [18]	18	Hemicolectomy *n* = 9 Total colectomy *n* = 4 Proctocolectomy *n* = 3 Sigmoid resection *n* = 1	IV lidocaine *n* = 9	IV lidocaine infusion 2 mg/minute if > 70 kg, or 1 mg/minute if < 70 kg, stopped on return of bowel function or day 5 postoperatively	Recovery—fentanyl 15–30 micrograms every 15 min, or morphine 2 mg every 20 min PRN	7
			Placebo *n* = 9	IV saline infusion throughout, stopped on return of bowel function or day 5 postoperatively	PCA morphine 2 mg, lockout time 10 min	
Kaba [19]	40	Right hemicolectomy *n* = 9 Left hemicolectomy *n* = 31	IV lidocaine *n* = 20	Lidocaine 1.5 mg/kg i.v. bolus at induction **THEN** by 2 mg/kg/h i.v. infusion **THEN** 1.33 mg/kg/h i.v. infusion postoperatively for 24 h	Paracetamol 1 g 30 min prior to end of surgery THEN every 6 h for first 24 h Ketorolac 30 mg IV every 8 h for first 24 h Piritramide [opioid] as rescue medication—1 mg, lockout 5 min After 24 h Paracetamol 1 g oral every 6 h Diclofenac 75 mg BD Tramadol 100 mg PRN	14
			Placebo *n* = 20	Normal saline i.v. bolus at induction **THEN** i.v. infusion of normal saline throughout procedure and postoperatively for 24 h		
Kim [20]	68	Right hemicolectomy *n* = 14 Left hemicolectomy *n* = 4 Anterior resection *n* = 49 Subtotal colectomy *n* = 1	IV lidocaine *n* = 32	IV bolus of lidocaine 1 mg/kg was given **THEN** IV infusion of lidocaine 1 mg/kg/h AND ketorolac 90 mg for 24 h	NSAIDs after ketorolac infusion stopped	14
			Placebo *n* = 36	5 ml bolus of normal saline **THEN** 90 mg ketorolac in 240 ml saline for 24 h		
Tikuisis [21]	60	Laparoscopic anterior resection *n* = 60	IV lidocaine *n* = 30	IV bolus of lidocaine 1.5 mg/kg was given [maximum 100 mg] **THEN** IV infusion of lidocaine 2 mg/kg/h during the entire surgical procedure **THEN** 1 mg/kg/h in the postoperative anaesthesia care unit and continued for the first 24 h after surgery	Fentanyl 24 h post op infusion 0.1 µg/kg/hr Ketorolac 30 mg PRN	12
			Placebo *n* = 30	IV bolus of normal saline **THEN** continuous infusion of normal saline during surgery and for 24 h after the operation		
			Placebo *n* = 20	Normal saline via IV infusion and epidural throughout procedure		
Open						
Kuo [22]	60	‘Elective surgery for colon cancer’, procedure not further detailed	IV lidocaine *n* = 20	Lidocaine 2 mg/kg i.v. infusion over 10 min prior to induction **THEN** 3 mg/kg/hr i.v. infusion for duration of procedure	Intraoperative: fentanyl 1 µg/kg Patient-controlled epidural analgesia [PCEA] postop with morphine 0.1 mg/ml in ropivacaine 0.2%. 4 ml bolus 15 min lockout	13
			Placebo *n* = 20	Normal saline via IV infusion and epidural throughout procedure		
Herroeder [23]	60	Ileocaecal resection *n* = 2 Right hemicolectomy *n* = 10 Left hemicolectomy *n* = 5 Subtotal colectomy *n* = 1 Proctocolectomy *n* = 4 Sigmoid resection *n* = 20 Anterior resection *n* = 9 Other *n* = 9	IV lidocaine *n* = 31	Lidocaine 1.5 mg/kg i.v. bolus at induction **THEN** 2 mg/min i.v. infusion during procedure terminated 4 h after skin closure	IV piritramide 30 min prior to end of procedure PCA 2 mg piritramide PRN, lockout of 10 min 1 g metamizol or 1 g paracetamol 6-hourly	15
			Placebo *n* = 29	Saline i.v. bolus at induction **THEN** i.v. infusion during procedure terminated 4 h after skin closure		
Staikou [24]	60	Right hemicolectomy * n* = 12 Left hemicolectomy * n* = 8 Sigmoidectomy * n* = 29 Anterior resection * n* = 5 Abdominoperineal resection * n* = 6	IV lidocaine * n* = 20	Bolus of 1.5 mg/kg lidocaine IV **THEN** continuous IV infusion at 2 mg/kg/hr, stopped at end of procedure	At start of skin suturing 20 mg ropivacaine and 1 mg morphine administered epidurally to all patients Intraoperative Remifentanil PCA with ropivacaine 2 mg/ml and morphine 0.1 mg/ml—released 4 ml per delivery, lockout of 20 min Up to 4 g paracetamol + 16 mg lornoxicam per day	14
			Placebo* n* = 20	Saline infusion IV and epidurally at volumes and rates as if containing lidocaine, stopped at end of procedure		
Harvey [11]	22	‘Elective bowel surgery’, procedure not further detailed	IV lidocaine *n* = 11	IV infusion postoperatively only of lidocaine 1 mg/min for 24 h	Morphine PCA	12
			Placebo *n* = 11	Saline infusion 10 ml/hr postoperatively for 24 h		
Ho [25]	58	Stoma formation *n* = 28 Anterior resection *n* = 24 Right hemicolectomy *n* = 14 Left hemicolectomy *n* = 2 Total colectomy *n* = 5 Abdominoperineal resection *n* = 4 Proctocolectomy *n* = 4 Other *n* = 4	IV lidocaine *n* = 28	1.5 mg/kg over 5 min (lidocaine 2.5%, 0.06 ml/kg) immediately postinduction, followed by 1 mg/kg/h (lidocaine 2.5%, 0.04 ml/kg/h) for 48 h	IV fentanyl PCA Other multimodal agents given at the discretion of the anaesthetist	14
			Placebo *n* = 29	Equal volume, rate and duration of infusion of normal saline		

*RCT* randomized controlled trial, *IV* intravenous, *PRN* as required, *IM* intramuscular, *PR* per rectum, *PO* oral, *PCA* patient-controlled analgesia, *NSAID* non-steroidal anti-inflammatory drug, *SC* subcutaneous, *IP* intraperitoneal

### Statistical analysis

Data were analyzed using the mean weighted difference (WMD) and pooled odds ratios for continuous variables and dichotomous data, respectively. A random effects model was selected on the basis of radial plots of the primary outcome. Statistical significance was set at *p* < 0.05. Heterogeneity was classified as low (< 33%), medium (33–66), or high (> 66%) using I2 estimated using the restricted maximum likelihood estimator function. Data were analyzed using the *metafor* package in R (version 3.4.2, R statistical programming, Vienna) [26].

## Results

**Fig. 1:**
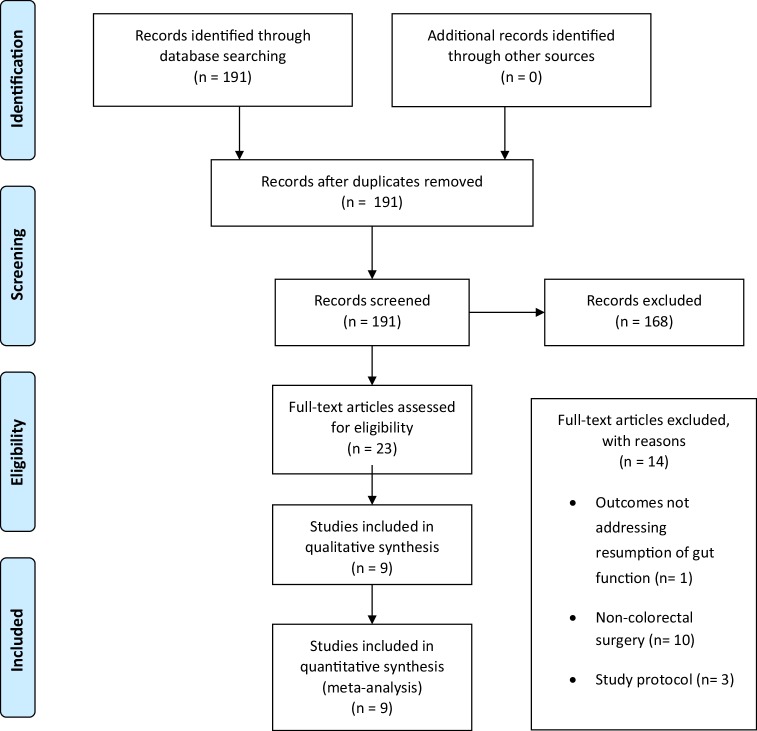
PRISMA flow diagram of search strategy and included studies

Nine RCTs were identified by the literature search strategy detailed in the Preferred Reporting Items for Systematic Reviews and Meta-analyses (PRISMA) flow diagram in Fig. 1, with a total of 405 patients. All results and figures are presented in supplementary data.

**Fig. 2:**
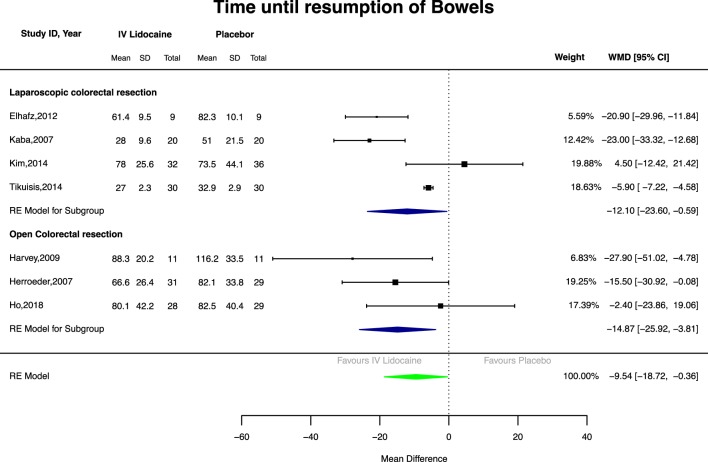
Forest plot of time from operation to first bowel movement

### Primary outcome: time to first bowel movement

IV lidocaine was associated with a significantly reduced time to first bowel movement (Fig. 2) in pooled analysis compared with placebo (seven studies, 325 patients, WMD − 9.54 h, 95%CI 18.72 to − 0.36, *p* = 0.04). In subgroup analyses, IV lidocaine reduced time to first bowel movement in both open (three studies, 139 patients; WMD − 14.87 h, 95%CI 25.92 to − 3.81, *p* = 0.008) and laparoscopic surgery (four studies, 186 patients; WMD − 12.1 h, 95%CI 23.6 to − 0.59, *p* = 0.04).

### Secondary outcomes

#### Time to first passage of flatus

Intravenous lidocaine did not significantly improve time to first passage of flatus in pooled analysis (8 studies, 345 patients; WMD − 3.42 h, 95%CI 10.41–3.58, *p* = 0.339). There was a significant decrease in the time to first passage of flatus in the open subgroup (five studies, 229 patients; WMD − 7.07 h, 95%CI 13.58 to − 0.57, *p* = 0.033) but not the laparoscopic subgroup (three studies, 126 patients; WMD − 4.58 h, 95%CI − 18.22 to 9.07, *p* = 0.511).

#### Time to resumption of diet

Only three studies reported this endpoint. IV lidocaine did not significantly hasten the time to toleration of diet in pooled analysis (three studies, 188 patients; WMD—10.93 h, 95%CI − 23.03 to 1.17, *p* = 0.077). IV lidocaine was associated with a shorter time to toleration of diet compared with placebo only in the laparoscopic subgroup (two studies, 128 patients; WMD − 5.97 h, 95%CI − 6.88 to − 5.09, *p* < 0.001). However, this was heavily weighted by a single study. Furthermore, resumption of diet is a less objective measurement than return of bowel function, as it greatly varies by individual practice.

#### Nausea and vomiting

There was no significant difference in nausea and vomiting events when comparing IV lidocaine with placebo in pooled analysis (five studies, 271 patients, OR 0.54, 95%CI 0.21–1.41, *p* = 0.150). There was no significant difference in the laparoscopic and open subgroups.

#### Incidence of postoperative ileus

In pooled analysis, there was a significant reduction in the incidence of postoperative ileus in the IV lidocaine group (five studies, 256 patients, OR 0.32, 95%CI 0.15–0.71, *p* = 0.02). No differences in the incidence of postoperative ileus were seen in subgroup analyses.

#### Pain score at rest at 24 h

Intravenous lidocaine was associated with lower pain scores at rest at 24 h compared with placebo (seven studies, 280 patients, WMD − 0.72, 95%CI − 1.31 to − 0.13, *p* = 0.020). This benefit was seen in the open subgroup (four studies, 159 patients, WMD − 0.36, 95%CI − 0.66 to − 0.06, *p* = 0.02). There was no significant difference in the laparoscopic subgroup.

#### Pain score on movement at 24 h

Intravenous lidocaine was associated with lower pain scores on movement at 24 h compared with placebo (four studies, 133 patients, WMD − 1.02, CI − 1.89 to − 0.14, *p* = 0.020). This effect was seen in the laparoscopic (two studies, WMD − 1.70, 95%CI − 2.18 to − 1.22, *p* < 0.0001) but not the open subgroup (two studies, 80 patients, WMD − 0.38, 95%CI − 1.06 to 0.31, *p* = 0.28).

#### Opioid consumption during first 24 h after operation

There was no difference in opioid consumption in the first 24 h after operation in pooled or subgroup analyses (pooled analysis five studies, 205 patients; WMD − 4.24 mg, 95%CI − 9.86 to 1.38, *p* = 0.14).

#### Total opioid consumption

There was no significant difference in total opioid consumption in pooled or subgroup analyses (pooled analysis seven studies, 305 patients; WMD − 5.82 mg, 95%CI − 22.32 to 10.67, *p* = 0.49).

#### Length of stay

Intravenous lidocaine was associated with shorter length of stay in pooled analysis (seven studies, 347 patients; WMD − 17.84 h, 95%CI − 32.95 to − 2.74 h, *p* = 0.020). This was the case in both laparoscopic (three studies, 168 patients; WMD − 23.04 h, 95%CI − 32.52 to − 13.56 h, *p* < 0.0001) and open subgroups (four studies, 179 patients; WMD − 19.62 h, 95% CI − 36.66 to − 2.59 h, *p* = 0.020).

Forest plots for secondary outcomes are shown in Supplementary data 1.

## Discussion

Previous meta-analyses of perioperative IV lidocaine have included a diverse range of operative procedures and focused on opioid analgesic consumption and pain scores [27, 28]. This meta-analysis examined the effect of IV lidocaine on return of GI function after major colorectal surgery, a critical determinant of recovery and discharge from hospital for this patient group. Time to first bowel movement was reduced by approximately 15 h in open and 12 h in laparoscopic surgery. Consistent with this finding was a substantially reduced risk of postoperative ileus (OR 0.32), reduced early pain scores, and reduced length of hospital stay of approximately 18 h (95% CI − 2.74 to − 32.95 h). If these findings were replicated in routine practice, perioperative IV lidocaine could hasten recovery, reduce postoperative ileus, and reduce length of stay for a significant proportion of patients. Given that colectomy is a common operation undertaken in every acute hospital in the western world, considerable cost savings could be achieved in reduced bed occupancy from this straightforward and inexpensive intervention. Although this analysis did not specifically study the safety of IV lidocaine, it is a familiar drug and the previous reviews suggest a low incidence of IV lidocaine-associated toxicity [29].

The mechanism of action of IV lidocaine in this setting remains uncertain. Pain scores were lower with IV lidocaine, but opioid consumption was not significantly different, suggesting that the faster return of gut function was not solely due to opiate sparing [30, 31]. IV lidocaine has a variety of analgesic and anti-inflammatory effects mediated through sodium channel receptors (recently summarized in detail [32]) and is known to reduce postoperative serum cytokine levels, suggesting that it acts centrally and peripherally to blunt the pro-inflammatory response to surgery [18, 33]. Postoperative ileus is multifactorial, and IV lidocaine probably acts via more than one mechanism.

Our study aimed to highlight potential benefits of perioperative IV lidocaine to colorectal surgeons, but has several limitations and its results need to be interpreted carefully. No study reported the consensus-derived, validated GI-2 or GI-3 composite endpoints of GI function and not all univariate endpoints were reported by all studies (for example, time to resumption of diet, integral to the GI2/GI-3 endpoint, was reported by only three studies). Although dose was consistent between studies (1–2 mg/kg/h), duration of infusion was not: most studies used 24 h, but ranged from operation only [22, 24] to 5 days (Elhafz et al. [18]). The latter study is, therefore, a methodological outlier, elimination of which in sensitivity analysis (Supplementary data 2) leads to a loss of statistical significance for the laparoscopic subgroup for the primary endpoint. This sensitivity analysis shows that our results are susceptible to removal of individual studies, reflecting study heterogeneity and the small total sample size, and is another reason for cautious interpretation.

Currently, the ‘correct’ duration of infusion is unknown. The intraoperative period is probably the most important; thereafter, continuation of the infusion depends on availability of cardiac monitoring beyond the theatre suite, which may be dictated by local resources. Plasma accumulation, and hence risk of toxicity, is unlikely with less than 24 h continuous infusion [32]. The authors’ local practice is a 12-h infusion, and the UK ALLEGRO trial of perioperative IV lidocaine will compare outcomes from 6-h and 12-h infusion [34].

Finally, few studies reported a perioperative protocol consistent with modern enhanced recovery principles. Notably, those that did had short lengths of stay (median 3–4 days) and showed a clear benefit from IV lidocaine [19, 21]. In contrast, where enhanced recovery protocols were not used/reported and length of stay was longer (median 8–9 days), no benefit was shown [20, 25]. This suggests that IV lidocaine exerted the greatest benefit on early recovery and was most effective within a modern patient care protocol; conversely, where length of stay was long (outdated care pathways, complex case mix, or high complication rates), a benefit was more difficult to detect.

## Conclusions

Although this analysis reduces heterogeneity by including colorectal surgery only, most studies were small, set in contrasting perioperative care protocols and reported sub-optimal endpoints to assess postoperative GI function. Nevertheless, an intriguing signal of benefit from IV lidocaine was seen consistently across the reported outcomes, suggesting that perioperative IV lidocaine could have a clinically meaningful effect on return of GI function, and hence, length of stay after colorectal surgery. IV lidocaine is inexpensive, straightforward to administer within existing evidence-based perioperative care protocols, and appears safe. Large-scale pragmatic effectiveness trials embedded within modern perioperative protocols are warranted to confirm or refute these findings and optimize dose and duration of infusion.

## Electronic supplementary material

Below is the link to the electronic supplementary material.

Supplementary material 1 (PDF 268 KB)

Supplementary material  2 (PDF 192 KB)
