# Supplementary material for: Meta-analysis of the effect of perioperative intravenous lidocaine on return of gastrointestinal function after colorectal surgery
Source: Tech Coloproctol. 2019 Feb 5;23(1):15–24. doi: 10.1007/s10151-019-1927-1 (PMC6394718; doi:10.1007/s10151-019-1927-1)
Supplement: Supplementary file 1 — Supplementary material 1 (PDF 268 KB) [file 10151_2019_1927_MOESM1_ESM.pdf]

Colorectal IV lignocaine meta analysis

Nicholas Ventham

Sat Sep 1 18:07:39 2018

Contents

S1a Time until Bowels **2**

S1b Time until Flatus **4**

S1c Time until Diet **5**

S2 Nausea and Vomiting **6**

S3 Ileus **7**

S4 Pain scores at Rest at 24 hours **8**

S5 Pain scores on Movement at 24 hours **9**

S6 Opiate Comsumption Day One **10**

S7 Total Opiate Consumption **11**

S8 Length of Stay **12**

S9 Sensitivity Analysis 1; Time until Bowels, Infusion duration **13**

S1a Time until Bowels

Radial Plots

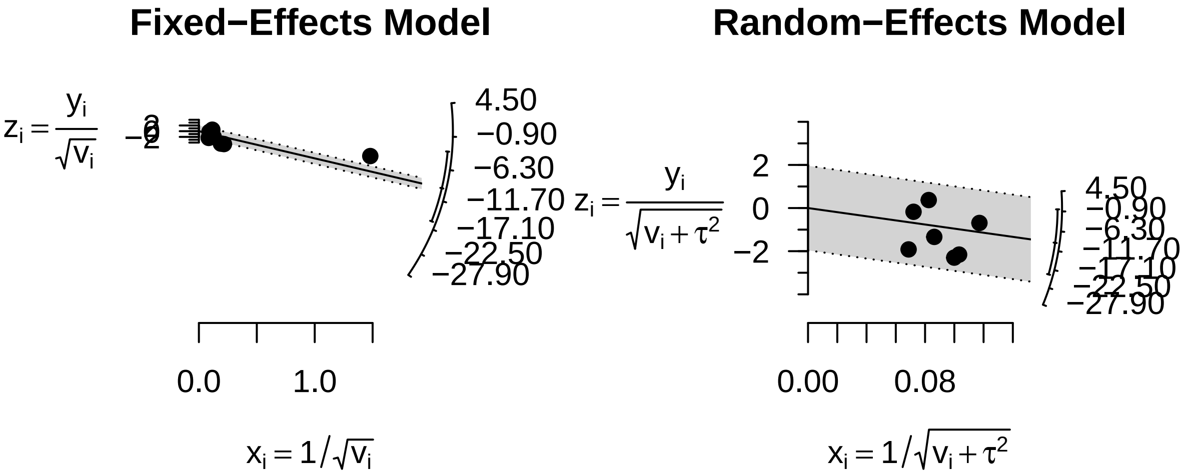

There were 7 studies with a total of 325 patients

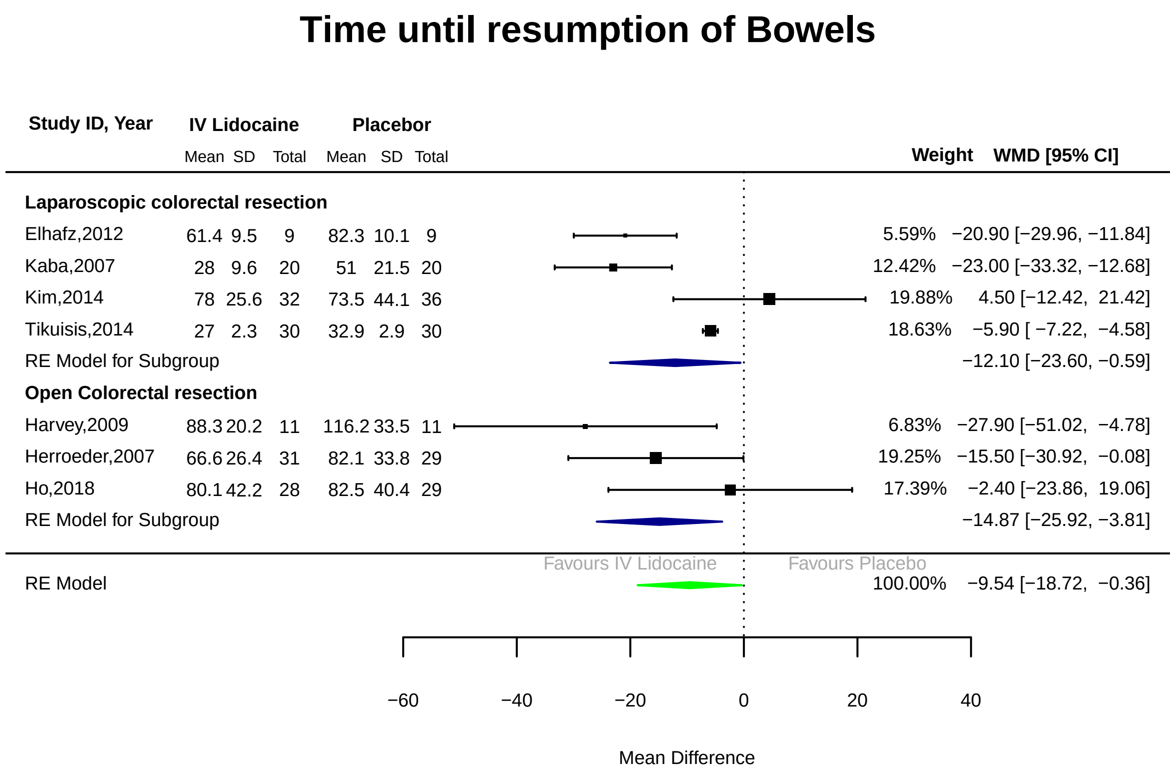

- **Bowels: Overall Results**
  - Hetrogeneity I2 = 74.89 %
  - WMD = -9.54
  - Lower CI = -18.72
  - Upper CI = -0.36
  - pValue = 0.04
  - Funnel Plot Asymmetry pValue = 0.88

- *Bowels: Open Surgery: IV Lidocaine Versus Placebo*
  - Hetrogeneity I2 = 0.66 %
  - WMD = -14.87
  - Lower CI = -25.92
  - Upper CI = -3.81
  - pValue = 0.008

- *Bowels: Laparoscopic Surgery: IV Lidocaine Versus Placebo*
  - Hetrogeneity I2 = 87.42 %
  - WMD = -12.1
  - Lower CI = -23.6
  - Upper CI = -0.59
  - pValue = 0.04

S1b Time until Flatus

There were 8 studies with a total of 345 patients

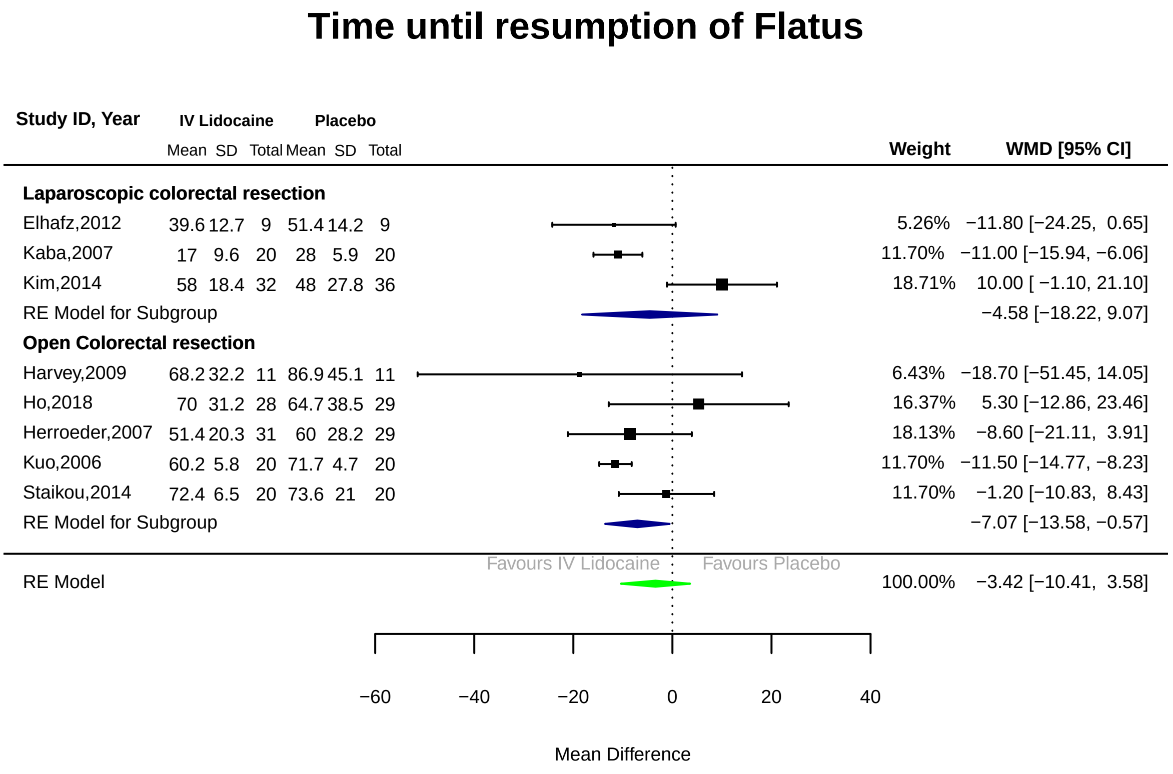

- **Flatus: Overall Results**
  - Hetrogeneity I2 = 72.11 %
  - WMD = -3.42
  - Lower CI = -10.41
  - Upper CI = 3.58
  - $pValue = 0.338666$
  - Funnel Plot Asymmetry pValue = 0.98

- *Flatus: Open Surgery: IV Lidocaine Versus Placebo*
  - Hetrogeneity I2 = 43.72 %
  - WMD = -7.07
  - Lower CI = -13.58
  - Upper CI = -0.57
  - $pValue = 0.0331305$

- *Flatus: Laparoscopic Surgery: IV Lidocaine Versus Placebo*
  - Hetrogeneity I2 = 84.43 %
  - WMD = -4.58
  - Lower CI = -18.22
  - Upper CI = 9.07
  - $pValue = 0.5109973$

S1c Time until Diet

There were 3 studies with a total of 188 patients

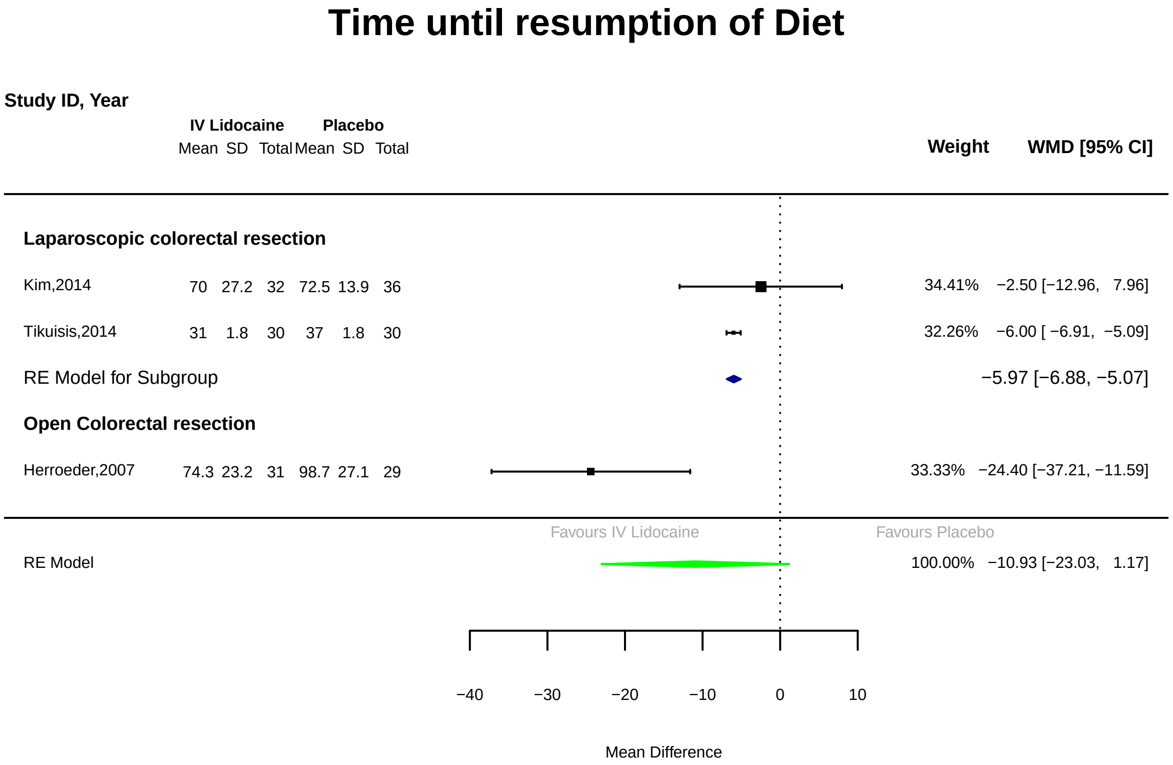

- **Diet: Overall Results**
  - Hetrogeneity I2 = 83.89 %
  - WMD = -10.93
  - Lower CI = -23.03
  - Upper CI = 1.17
  - pValue = 0.0766014
  - Funnel Plot Asymmetry pValue = 0.51

- *Diet: Laparoscopic Surgery: IV Lidocaine Versus Placebo*
  - Hetrogeneity I2 = 0 %
  - WMD = -5.97
  - Lower CI = -6.88
  - Upper CI = -5.07
  - $pValue = 4.3907807 \times 10^{-38}$

S2 Nausea and Vomiting

There were 5 studies with a total of 271 patients

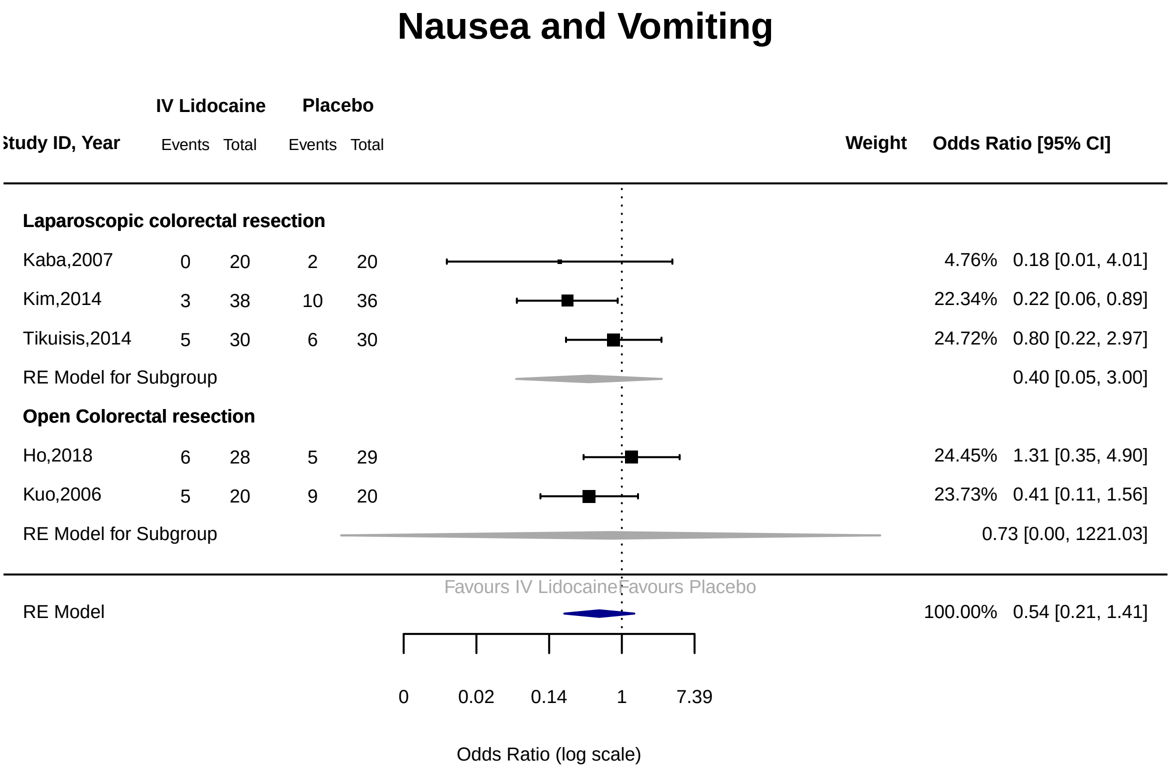

- **Nausea and vomiting: Overall Results**
  - Hetrogeneity I2 = 6.7 %
  - OR = 0.54
  - Lower CI = 0.21
  - Upper CI = 1.41
  - $pValue = 0.15$
  - Funnel Plot Asymmetry pValue = 0.48

- *Nausea and vomiting: Laparoscopic Surgery: IV Lidocaine Versus Placebo*
  - Hetrogeneity I2 = 0.38 %
  - OR = 0.4
  - Lower CI = 0.05
  - Upper CI = 3
  - $pValue = 0.19$

- *Nausea and vomiting: Open Surgery: IV Lidocaine Versus Placebo*
  - Hetrogeneity I2 = 32.3 %
  - OR = 0.73
  - Lower CI = 0
  - Upper CI = 1221.03
  - $pValue = 0.69$

S3 Ileus

There were 5 studies with a total of 256 patients

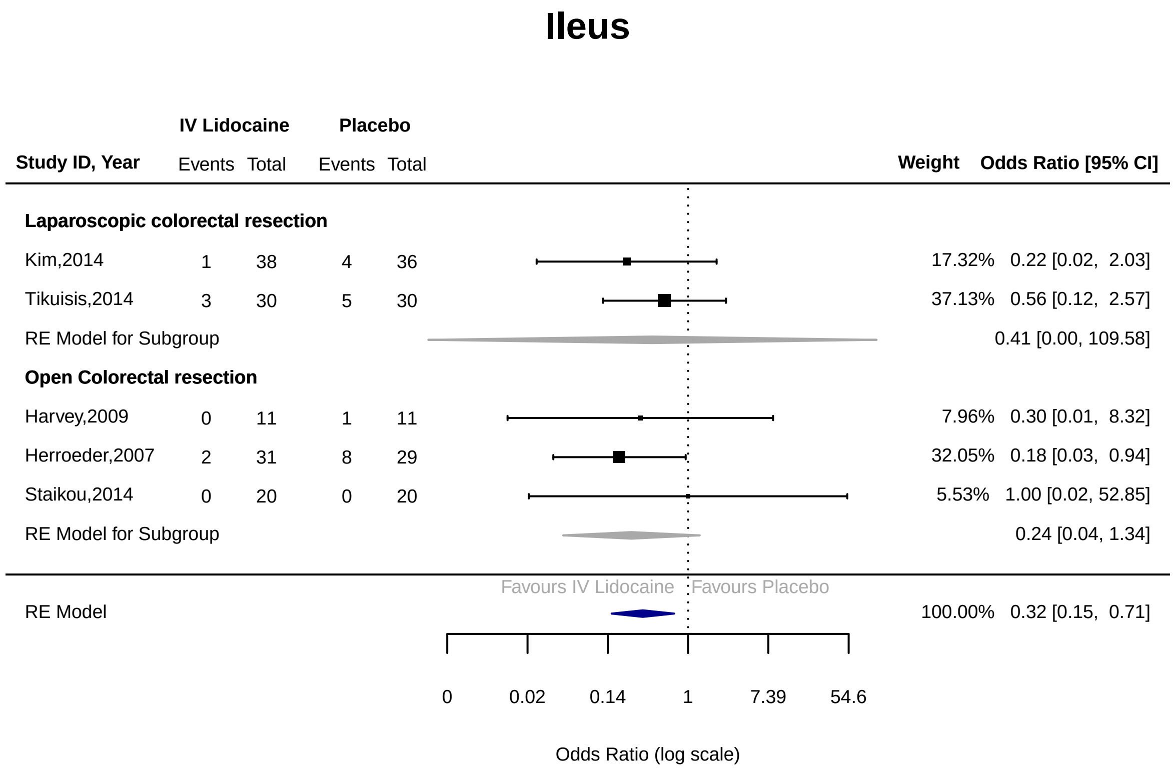

- **Ileus: Overall Results**
  - Hetrogeneity I2 = 0 %
  - OR = 0.32
  - Lower CI = 0.15
  - Upper CI = 0.71
  - $pValue = 0.02$
  - Funnel Plot Asymmetry pValue = 0.77

- *Ileus: Laparoscopic Surgery: IV Lidocaine Versus Placebo*
  - Hetrogeneity I2 = 0 %
  - OR = 0.41
  - Lower CI = 0
  - Upper CI = 109.58
  - $pValue = 0.29$

- *Ileus: Open Surgery: IV Lidocaine Versus Placebo*
  - Hetrogeneity I2 = 0 %
  - OR = 0.24
  - Lower CI = 0.04
  - Upper CI = 1.34
  - $pValue = 0.07$

S4 Pain scores at Rest at 24 hours

There were 7 studies with a total of 280 patients

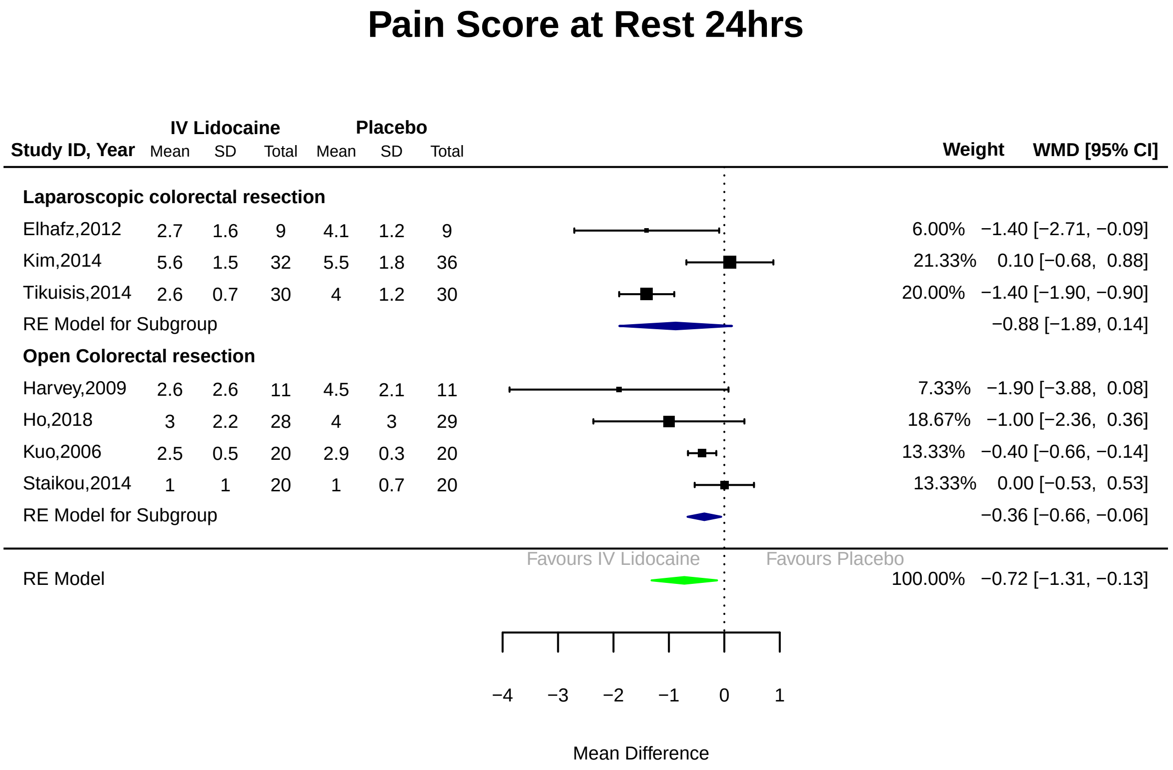

- **PainRest24hrs: Overall Results**
  - Hetrogeneity I2 = 77.03 %
  - WMD = -0.72
  - Lower CI = -1.31
  - Upper CI = -0.13
  - $pValue = 0.02$
  - Funnel Plot Asymmetry pValue = 0.28

- *PainRest24hrs: Open Surgery: IV Lidocaine Versus Placebo*
  - Hetrogeneity I2 = 14.45 %
  - WMD = -0.36
  - Lower CI = -0.66
  - Upper CI = -0.06
  - $pValue = 0.02$

- *PainRest24hrs: Laparoscopic Surgery: IV Lidocaine Versus Placebo*
  - Hetrogeneity I2 = 78.82 %
  - WMD = -0.88
  - Lower CI = -1.89
  - Upper CI = 0.14
  - $pValue = 0.09$

S5 Pain scores on Movement at 24 hours

There were 4 studies with a total of 133 patients

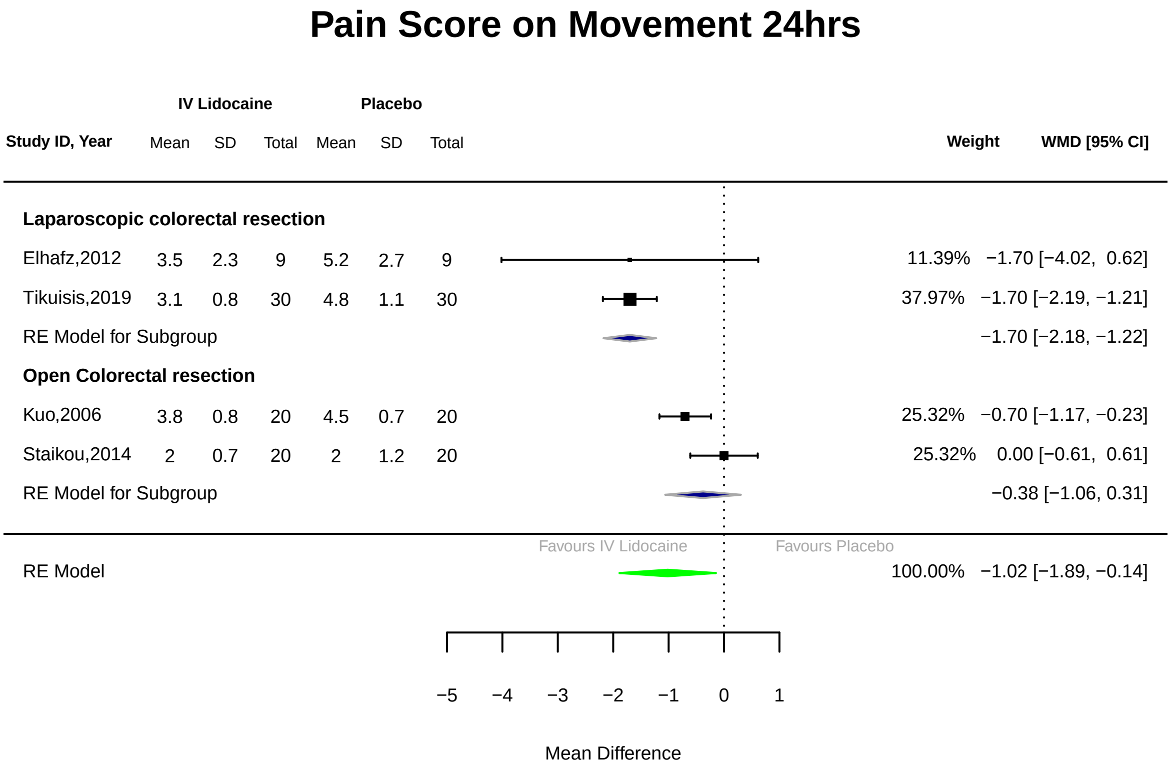

- **PainMove24hrs: Overall Results**
  - Hetrogeneity I2 = 84.85 %
  - WMD = -1.02
  - Lower CI = -1.89
  - Upper CI = -0.14
  - $pValue = 0.02$
  - Funnel Plot Asymmetry pValue = 0.69

- *PainMove24hrs: Open Surgery: IV Lidocaine Versus Placebo*
  - Hetrogeneity I2 = 68.78 %
  - WMD = -0.38
  - Lower CI = -1.06
  - Upper CI = 0.31
  - $pValue = 0.28$

- *PainMove24hrs: Laparoscopic Surgery: IV Lidocaine Versus Placebo*
  - Hetrogeneity I2 = 0 %
  - WMD = -1.7
  - Lower CI = -2.18
  - Upper CI = -1.22
  - $pValue = 2.65e - 12$

S6 Opiate Comsumption Day One

There were 5 studies with a total of 205 patients

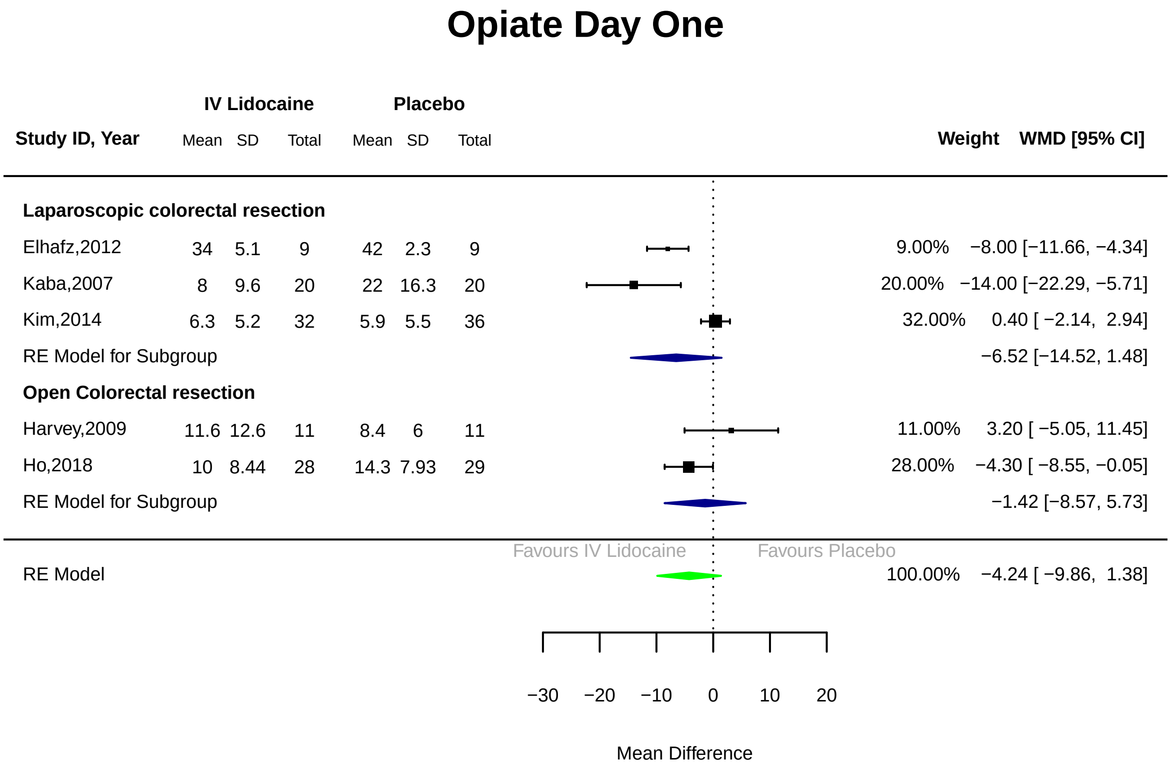

- **OpiateDayOne: Overall Results**
  - Hetrogeneity I2 = 84.83 %
  - WMD = -4.24
  - Lower CI = -9.86
  - Upper CI = 1.38
  - $pValue = 0.14$
  - Funnel Plot Asymmetry pValue = 0.36

- *OpiateDayOne: Laparoscopic Surgery: IV Lidocaine Versus Placebo*
  - Hetrogeneity I2 = 91.04 %
  - WMD = -6.52
  - Lower CI = -14.52
  - Upper CI = 1.48
  - $pValue = 0.11$

S7 Total Opiate Consumption

There were 7 studies with a total of 305 patients

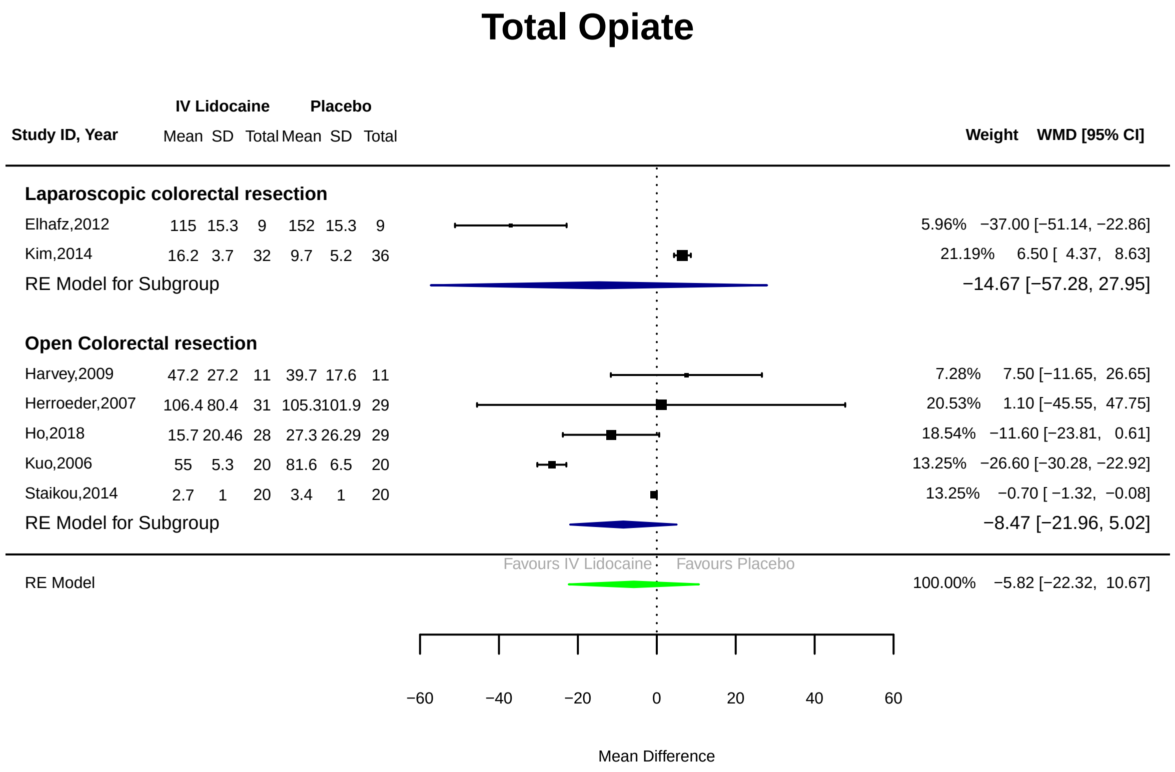

- **TotalOpiate: Overall Results**
  - Hetrogeneity I2 = 98.99 %
  - WMD = -5.82
  - Lower CI = -22.32
  - Upper CI = 10.67
  - $pValue = 0.49$
  - Funnel Plot Asymmetry pValue = 0.84

- *TotalOpiate: Open Surgery: IV Lidocaine Versus Placebo*
  - Hetrogeneity I2 = 96.47 %
  - WMD = -8.47
  - Lower CI = -21.96
  - Upper CI = 5.02
  - $pValue = 0.22$

- *TotalOpiate: Laparoscopic Surgery: IV Lidocaine Versus Placebo*
  - Hetrogeneity I2 = 97.19 %
  - WMD = -14.67
  - Lower CI = -57.28
  - Upper CI = 27.95
  - $pValue = 0.5$

S8 Length of Stay

There were 7 studies with a total of 347 patients

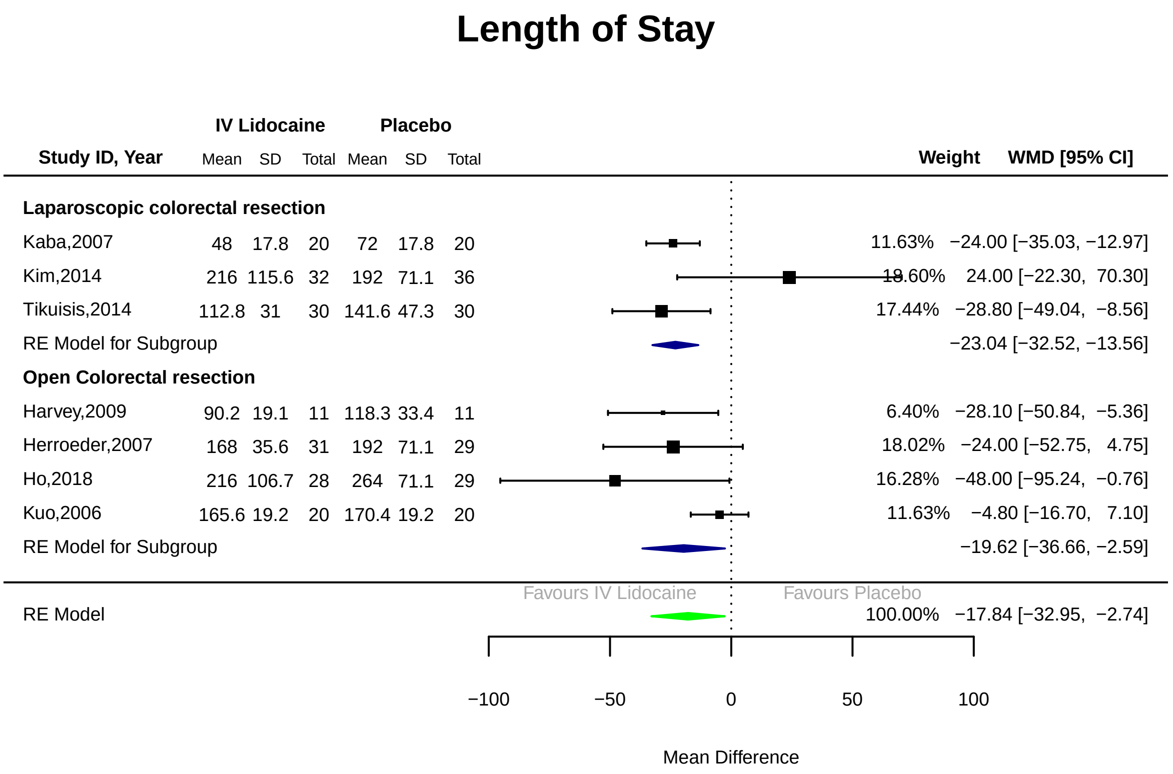

- **LoS: Overall Results**
  - Hetrogeneity I2 = 45.94 %
  - WMD = -17.84
  - Lower CI = -32.95
  - Upper CI = -2.74
  - $pValue = 0.02$
  - Funnel Plot Asymmetry pValue = 0.66

- *LoS: Open Surgery: IV Lidocaine Versus Placebo*
  - Hetrogeneity I2 = 50.33 %
  - WMD = -19.62
  - Lower CI = -36.66
  - Upper CI = -2.59
  - $pValue = 0.02$

- *LoS: Laparoscopic Surgery: IV Lidocaine Versus Placebo*
  - Hetrogeneity I2 = 0 %
  - WMD = -23.04
  - Lower CI = -32.52
  - Upper CI = -13.56
  - $pValue = 1.91e-06$

S9 Sensitivity Analysis 1; Time until Bowels, Infusion duration

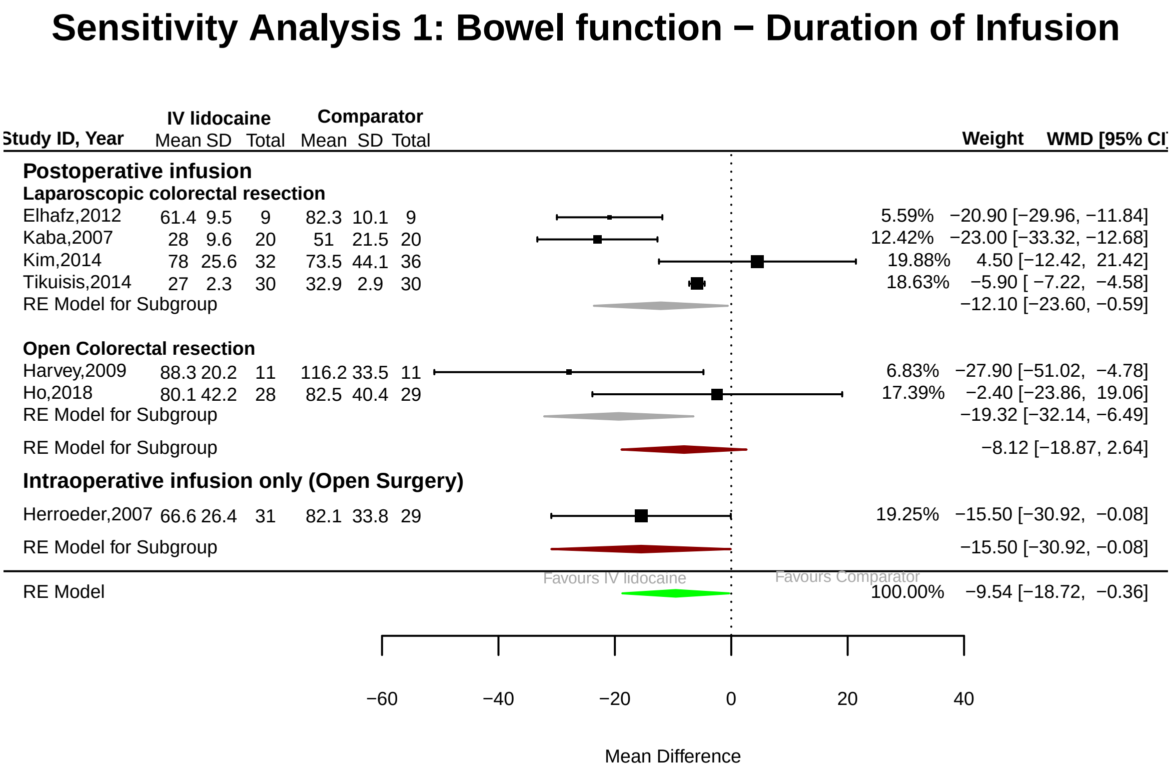

- *Time until Bowels: Post-op infusion*
  - Hetrogeneity I2 = 80.15 %
  - WMD = -8.12
  - Lower CI = -18.87
  - Upper CI = 2.64
  - pValue = 0.139

- *Time until Bowels: Intra-Op infusion only*
  - Hetrogeneity I2 = 0 %
  - WMD = -15.5
  - Lower CI = -30.92
  - Upper CI = -0.08
  - pValue = 0.049

“‘
