# Supplementary material for: Meta-analysis of the effect of perioperative intravenous lidocaine on return of gastrointestinal function after colorectal surgery
Source: Tech Coloproctol. 2019 Feb 5;23(1):15–24. doi: 10.1007/s10151-019-1927-1 (PMC6394718; doi:10.1007/s10151-019-1927-1)
Supplement: Supplementary file 2 — Supplementary material 2 (PDF 192 KB) [file 10151_2019_1927_MOESM2_ESM.pdf]

Sensitivity Analysis: Time to BO excluding each study

nventham

Fri Nov 30 09:56:06 2018

Contents

S1 Time until Bowels Excluding Elhafz 2

S2 Time until Bowels Excluding Kaba 3

S3 Time until Bowels Excluding Kim 4

S4 Time until Bowels Excluding Tikuisis 5

S5 Time until Bowels Excluding Harvey 6

S6 Time until Bowels Excluding Herroeder 7

S7 Time until Bowels Excluding Ho 8

S1 Time until Bowels Excluding Elhafz

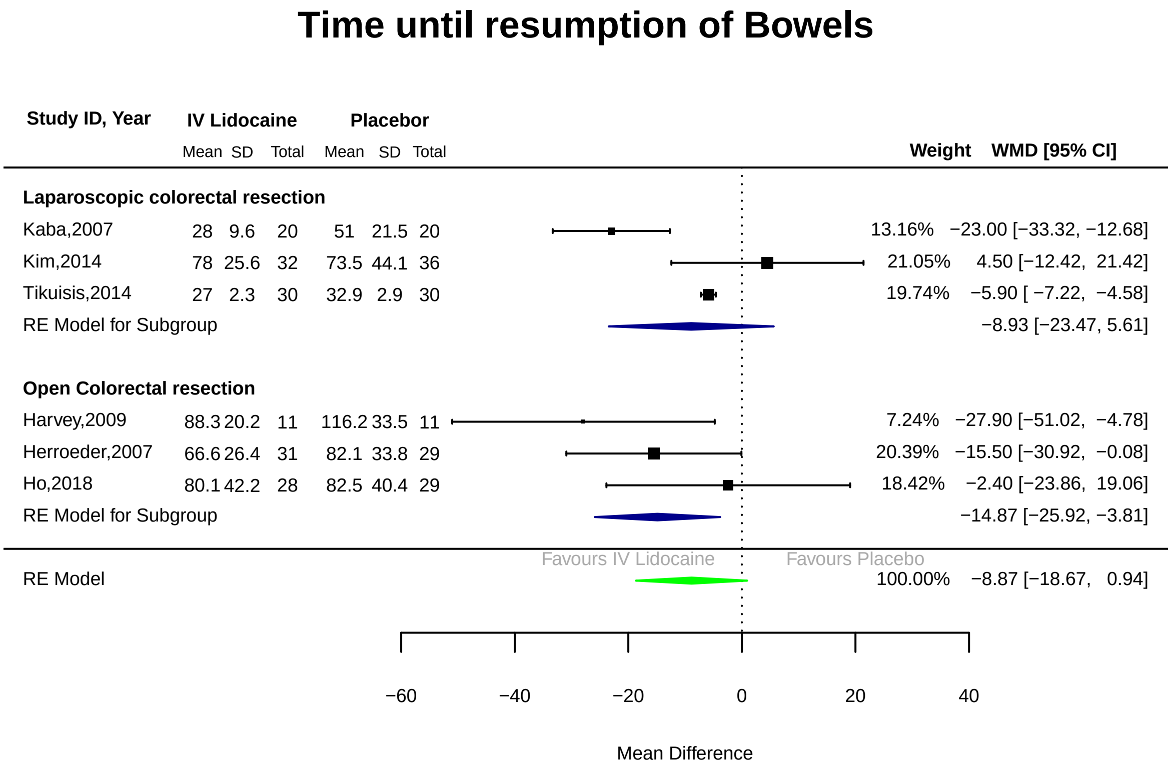

- **Bowels: Overall Results**
  - Hetrogeneity I2 = 70.79 %
  - WMD = -8.87
  - Lower CI = -18.67
  - Upper CI = 0.94
  - pValue = 0.08
  - Funnel Plot Asymmetry pValue = 0.96

- *Bowels: Open Surgery: IV Lidocaine Versus Placebo*
  - Hetrogeneity I2 = 0.66 %
  - WMD = -14.87
  - Lower CI = -25.92
  - Upper CI = -3.81
  - pValue = 0.008

- *Bowels: Laparoscopic Surgery: IV Lidocaine Versus Placebo*
  - Hetrogeneity I2 = 86.87 %
  - WMD = -8.93
  - Lower CI = -23.47
  - Upper CI = 5.61
  - pValue = 0.23

S2 Time until Bowels Excluding Kaba

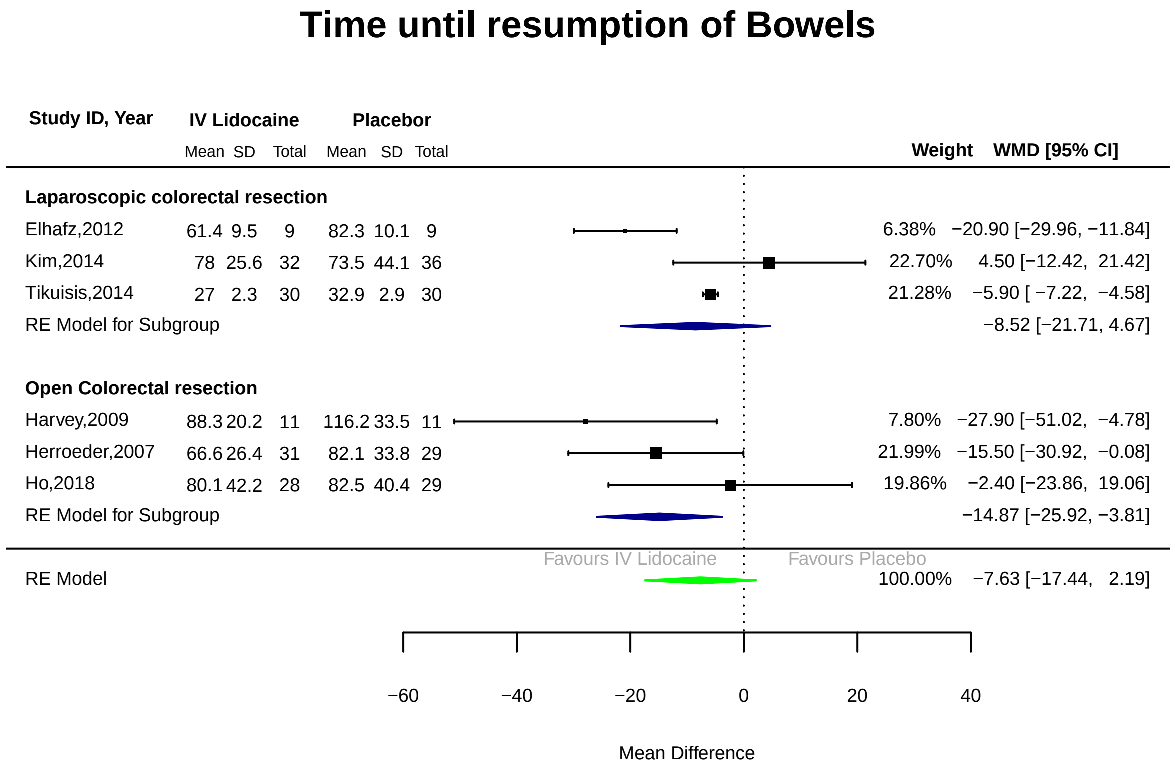

- **Bowels: Overall Results**
  - Hetrogeneity I2 = 69.79 %
  - WMD = -7.63
  - Lower CI = -17.44
  - Upper CI = 2.19
  - pValue = 0.13
  - Funnel Plot Asymmetry pValue = 0.97

- *Bowels: Open Surgery: IV Lidocaine Versus Placebo*
  - Hetrogeneity I2 = 0.66 %
  - WMD = -14.87
  - Lower CI = -25.92
  - Upper CI = -3.81
  - pValue = 0.008

- *Bowels: Laparoscopic Surgery: IV Lidocaine Versus Placebo*
  - Hetrogeneity I2 = 86.63 %
  - WMD = -8.52
  - Lower CI = -21.71
  - Upper CI = 4.67
  - pValue = 0.21

S3 Time until Bowels Excluding Kim

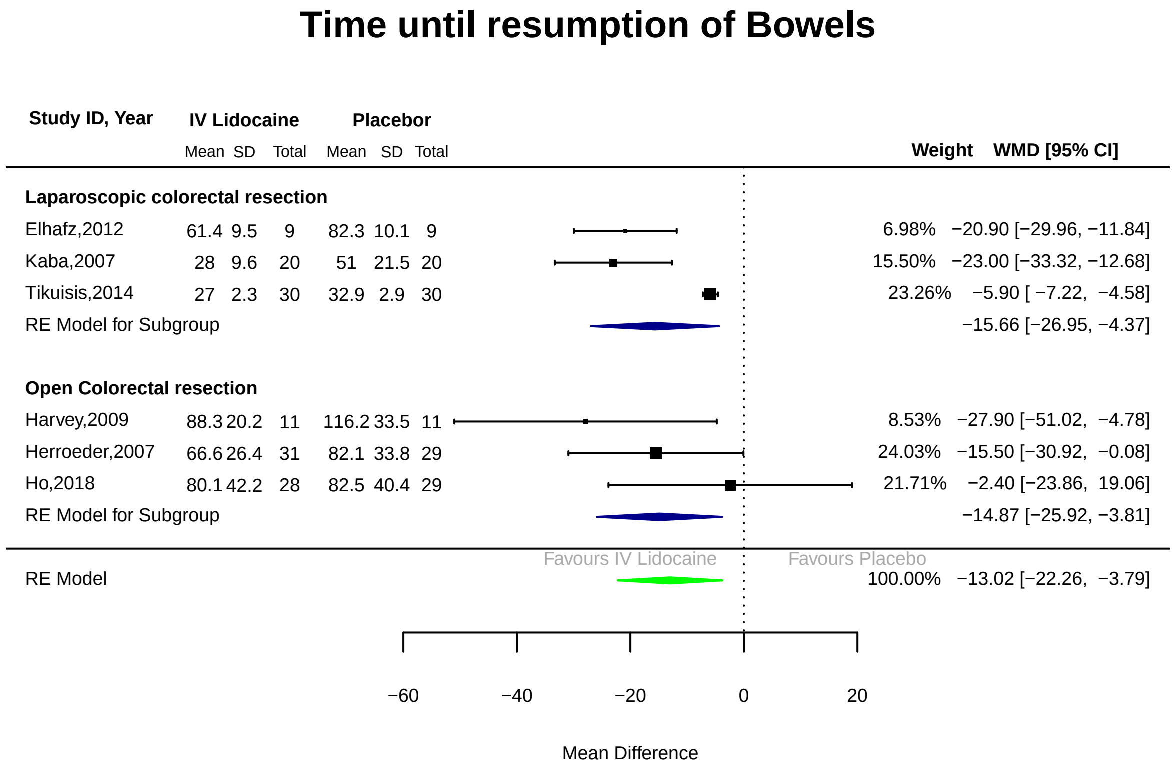

- **Bowels: Overall Results**
  - Hetrogeneity I2 = 71.76 %
  - WMD = -13.02
  - Lower CI = -22.26
  - Upper CI = -3.79
  - pValue = 0.01
  - Funnel Plot Asymmetry pValue = 0.82

- *Bowels: Open Surgery: IV Lidocaine Versus Placebo*
  - Hetrogeneity I2 = 0.66 %
  - WMD = -14.87
  - Lower CI = -25.92
  - Upper CI = -3.81
  - pValue = 0.008

- *Bowels: Laparoscopic Surgery: IV Lidocaine Versus Placebo*
  - Hetrogeneity I2 = 87.19 %
  - WMD = -15.66
  - Lower CI = -26.95
  - Upper CI = -4.37
  - pValue = 0.01

S4 Time until Bowels Excluding Tikuisis

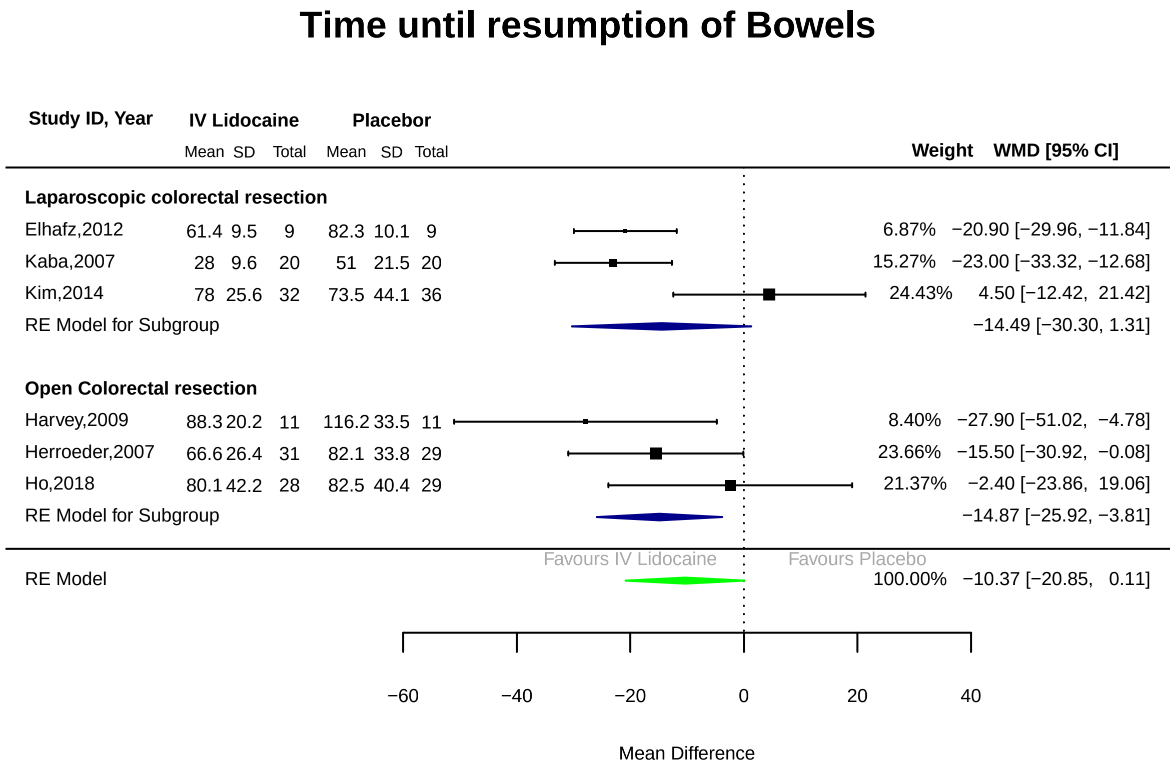

- **Bowels: Overall Results**
  - Hetrogeneity I2 = 56.91 %
  - WMD = -10.37
  - Lower CI = -20.85
  - Upper CI = 0.11
  - pValue = 0.05
  - Funnel Plot Asymmetry pValue = 0.32

- *Bowels: Open Surgery: IV Lidocaine Versus Placebo*
  - Hetrogeneity I2 = 0.66 %
  - WMD = -14.87
  - Lower CI = -25.92
  - Upper CI = -3.81
  - pValue = 0.008

- *Bowels: Laparoscopic Surgery: IV Lidocaine Versus Placebo*
  - Hetrogeneity I2 = 81.99 %
  - WMD = -14.49
  - Lower CI = -30.3
  - Upper CI = 1.31
  - pValue = 0.07

S5 Time until Bowels Excluding Harvey

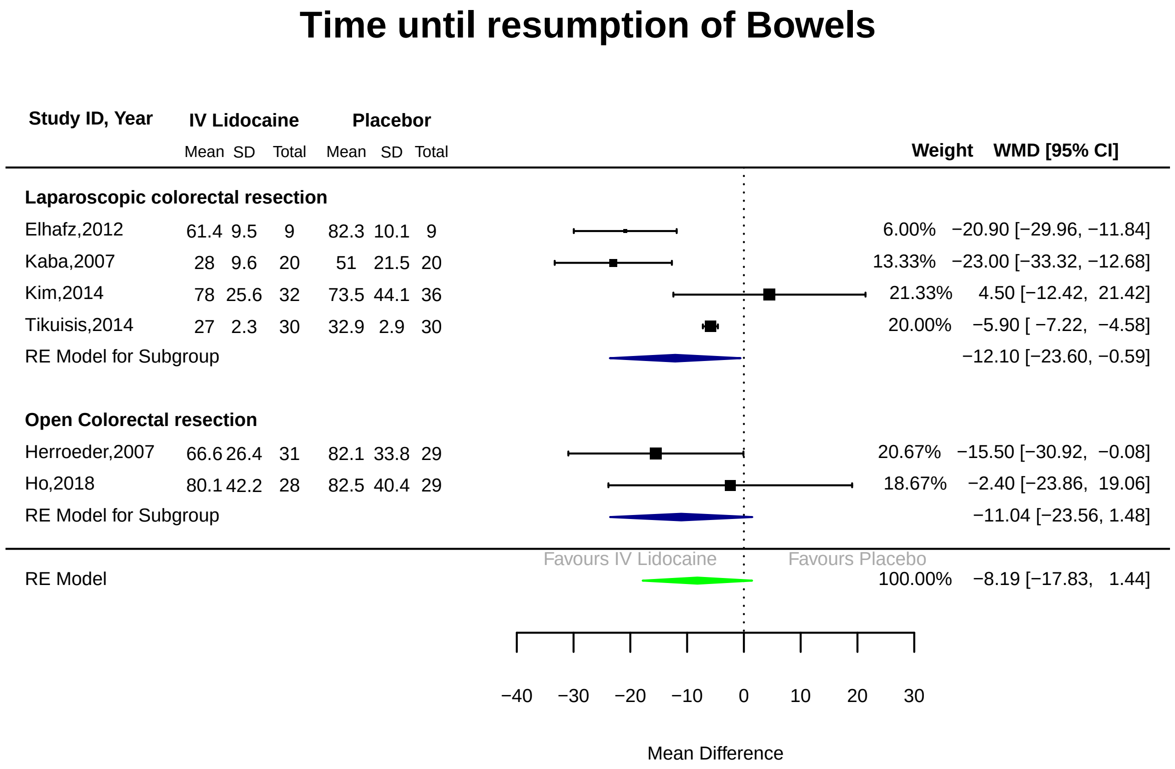

- **Bowels: Overall Results**
  - Hetrogeneity I2 = 77.19 %
  - WMD = -8.19
  - Lower CI = -17.83
  - Upper CI = 1.44
  - pValue = 0.1
  - Funnel Plot Asymmetry pValue = 0.6

- *Bowels: Open Surgery: IV Lidocaine Versus Placebo*
  - Hetrogeneity I2 = 0 %
  - WMD = -11.04
  - Lower CI = -23.56
  - Upper CI = 1.48
  - pValue = 0.084

- *Bowels: Laparoscopic Surgery: IV Lidocaine Versus Placebo*
  - Hetrogeneity I2 = 87.42 %
  - WMD = -12.1
  - Lower CI = -23.6
  - Upper CI = -0.59
  - pValue = 0.04

S6 Time until Bowels Excluding Herroeder

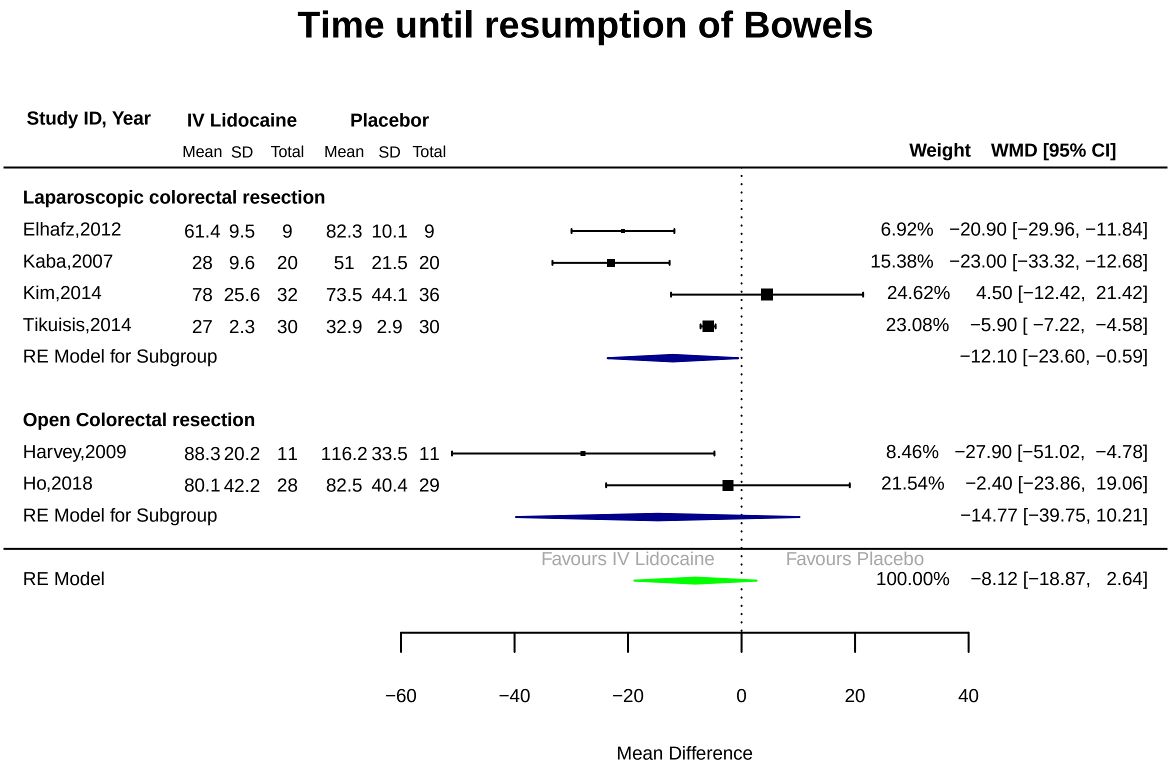

- **Bowels: Overall Results**
  - Hetrogeneity I2 = 80.15 %
  - WMD = -8.12
  - Lower CI = -18.87
  - Upper CI = 2.64
  - pValue = 0.14
  - Funnel Plot Asymmetry pValue = 0.84

- *Bowels: Open Surgery: IV Lidocaine Versus Placebo*
  - Hetrogeneity I2 = 60.17 %
  - WMD = -14.77
  - Lower CI = -39.75
  - Upper CI = 10.21
  - pValue = 0.246

- *Bowels: Laparoscopic Surgery: IV Lidocaine Versus Placebo*
  - Hetrogeneity I2 = 87.42 %
  - WMD = -12.1
  - Lower CI = -23.6
  - Upper CI = -0.59
  - pValue = 0.04

S7 Time until Bowels Excluding Ho

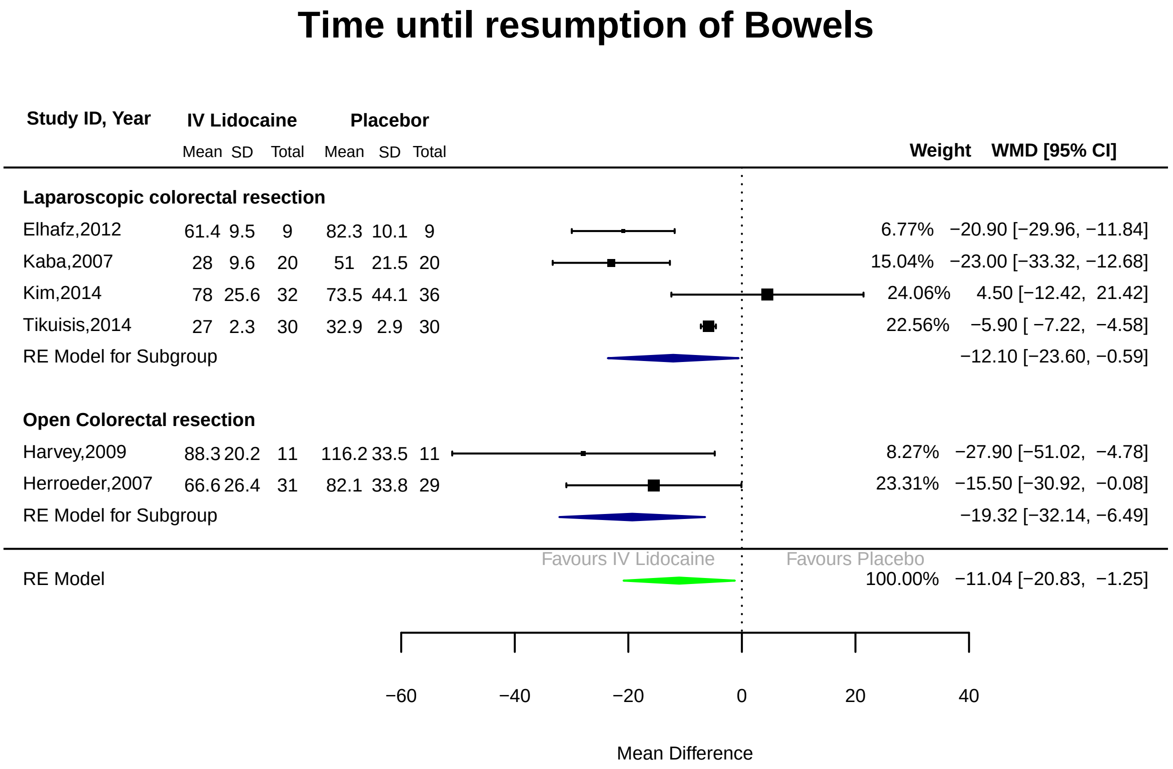

- **Bowels: Overall Results**
  - Hetrogeneity I2 = 78.54 %
  - WMD = -11.04
  - Lower CI = -20.83
  - Upper CI = -1.25
  - pValue = 0.03
  - Funnel Plot Asymmetry pValue = 0.81

- *Bowels: Open Surgery: IV Lidocaine Versus Placebo*
  - Hetrogeneity I2 = 0 %
  - WMD = -19.32
  - Lower CI = -32.14
  - Upper CI = -6.49
  - pValue = 0.003

- *Bowels: Laparoscopic Surgery: IV Lidocaine Versus Placebo*
  - Hetrogeneity I2 = 87.42 %
  - WMD = -12.1
  - Lower CI = -23.6
  - Upper CI = -0.59
  - pValue = 0.04

“‘
